# Cholesterol re-organisation and lipid de-packing by arginine-rich cell penetrating peptides: Role in membrane translocation

**DOI:** 10.1371/journal.pone.0210985

**Published:** 2019-01-23

**Authors:** Claudia Almeida, Ofelia Maniti, Margherita Di Pisa, Jean-Marie Swiecicki, Jesus Ayala-Sanmartin

**Affiliations:** CNRS, Sorbonne Université, École Normale Supérieure, Université PSL, Laboratoire des Biomolécules, Paris, France; Institut Curie, FRANCE

## Abstract

Cell penetrating peptides (CPPs) are able to transport hydrophilic molecules inside cells. To reach the cytosol, the peptide associated with a cargo must cross the plasma or the endosomal membrane. Different molecular mechanisms for peptide internalisation into cells have been proposed and it is becoming clear that the cellular internalisation mechanisms are different depending on the peptide sequence and structure and the target membrane. Herein, the penetration of three peptides into large unilamellar vesicles were studied: the homeodomain derived 16-residues penetratin, nona-arginine (R9), and a small peptide containing 6 arginine and 3 tryptophan residues (RW9). The membrane models were composed of phospholipids from natural sources containing different molecular species. We observed that among the three peptides, only the amphipathic peptide RW9 was able to cross the membrane vesicles in the liquid disordered state. The changes in the distribution of the previously characterized cholesterol-pyrene probe show that cholesterol-pyrene molecules dissociate from clusters upon membrane interaction with the three peptides and that the cholesterol environment becomes more disordered in the presence of RW9. Finally, we studied the effect of the peptides on lipid ordering on giant plasma membrane vesicles. The amphipathic peptides RW9 and its longer homologue RW16 induced lipid de-packing in plasma membrane vesicles. Overall, the data suggest that a disordered membrane favours the translocation of RW9, that the membrane cholesterol is redistributed during peptide interaction, and that the peptide amphipathic character is important to increase membrane fluidity and peptide membrane translocation.

## Introduction

Cell penetrating peptides (CPPs) are potential therapeutic vectors used to introduce molecules into mammalian cells, from small active molecules to siRNAs and DNA oligonucleotides. They are frequently enriched in arginine and lysine residues. For reviews see [1–3]. The molecular mechanisms involved in cellular membrane translocation of CPPs depend on peptide sequence, structure, concentration and also the nature of the cargo [2,4,5]. Several plasma membrane or endosomal membrane crossing mechanisms have been proposed which include the electroporation-like process [6], the neutralisation charges by guanidinium-phosphate hydrogen bonds [7], membrane inverted micelles formation [8,9], pore formation [10] and also direct translocation through the membrane [11].

Membrane models have been widely used to characterize the factors involved in peptide-membrane interactions. Different cell penetrating peptides have been shown to translocate inside giant and large unilamellar vesicles (GUVs and LUVs) [12–14]. CPPs induce different membrane perturbations such as changes in bilayer thickness [15,16] and induction of negative and positive curvature with the consequent formation of membrane tubes and invaginations [15,17–19]. They can also increase membrane flexibility [20] and change membrane lipid packing [21–23].

In all cases, the crucial step during CPP translocation into the cytosol is the crossing of the phospholipid bilayer. Prior to crossing the cell or endosomal membranes, CPPs interact with membrane phospholipids. This interaction can induce peptide to change conformation, to oligomerize, or affect the physicochemical properties of membranes. Those phenomena being keys during internalization, it is necessary to characterize the various effects of CPPs on membranes together with their propensity to spontaneously translocate through the membranes.

Cellular membranes are composed of different lipid species that are organized in a diversity of nano- and micro-domains [24–26]. Among membrane components, cholesterol (Chol) is a lipid that participates in the organization of membranes and leads to the formation of membrane domains such as the liquid ordered domains (“rafts”) [27,28]. These cholesterol rich domains are not exclusively composed of sphingomyelin and cholesterol but are also enriched in different species of saturated phospholipids [29,30].

Membrane lipid composition and membrane domains are supposed to play a role during membrane crossing of CPPs. Several reports showed that CPPs are able to induce membrane domains separation [16,21,23] and change lipid order [31]. It has been demonstrated that penetratin binding to model membranes increases with cholesterol [32]. Experiments with GUVs showed that the internalisation of nona-arginine peptide (R9) depends on the presence of the charged lipid phosphatidylglycerol and that the presence of cholesterol reduces the internalisation of R9 [33]. The depletion of cholesterol in leukaemia cells and in giant plasma membrane vesicles (GPMVs) results in an increase of oligo-arginines (R8, R9) and penetratin uptake [34–37].

In this paper we first studied the penetration of three well known and efficient CPPs into large unilamellar vesicles (LUVs); the homeodomain derived 16-residues penetratin, nona-arginine (R9), and a small peptide containing 6 arginine and 3 tryptophan residues which is able to form an amphipathic α-helix (RRWWRRWRR, RW9). To better mimic the cell membrane, we used phospholipids from natural sources containing different molecular species. Next, we followed the effect of CPP binding on cholesterol-pyrene distribution, a cholesterol probe previously characterized [38,39]. Finally, we studied the effect of CPPs on lipid packing on giant plasma membrane vesicles form the epithelial cell line MDCK. The results show that only RW9 was able to cross the membrane of LUVs and that the translocation only occurs when membranes are in a liquid disordered state. We show for the first time that cholesterol-pyrene disaggregates upon interaction with the three peptides and that the cholesterol environment becomes more disordered in the presence of RW9. We finally showed that the amphipathic peptides RW9 and RRWRRWWRRWWRRWRR (RW16) induce lipid de-packing in giant plasma membrane vesicles.

## Materials and methods

### Materials

Egg L-α-phosphatidylcholine (PC), egg sphingomyelin (SM), cholesterol (Chol), Melittin and dithionite (S_2_O_4_^2-^) were purchased from Sigma. Pyrene-labelled cholesterol (Py-met-chol) was a kind gift of Dr. André Lopez (Toulouse, France). di-4-ANEPPDHQ (ANE) was obtained from Dr Leslie M. Loew (Connecticut, USA). Penetratin (RQIKIWFQNRRMKWKK-NH2), Nona-arginine (R9), RW9 (RRWWRRWRR) and RW16 (RRWRRWWRRWWRRWRR) were synthesised and purified by HPLC as described [40]. NBD-labelling of peptides was performed as described in [12].

### Large unilamellar vesicles (LUVs)

Large unilamellar vesicles (100 nm diameter LUVs) were prepared as described [41]. Briefly, the appropriate amounts of lipids in a mixture of chloroform/methanol, 4/1 (v/v), were subjected to solvent evaporation. 500 μl of buffer were added to obtain a 1 mg ml^-1^ LUVs suspension. LUVs were obtained by extrusion through 100 nm polycarbonate membranes in 0.5 mM HEPES buffer (pH 7.4). For the Py-met-chol fluorescence experiments, the probe was added to the lipid mixture at 3.6% lipid molar ratio. The LUVs used in this study were PC, SM/Chol (1/1) and PC/SM/Chol (1/1/1) molar ratios.

### Peptide internalisation into LUVs

Peptide internalisation into LUVs was performed as published [12] with minor modification. LUVs (10 μM lipid content) and NBD-labelled peptides (0.1 μM) were incubated in a spectroscopic polystyrene cell at the desired temperature and the NBD fluorescence was followed by excitation at 460 nm and emission at 555 nm. We followed the fluorescence intensity variations with a FP 8300 Jasco fluorimeter. The experiments started observing the total peptide fluorescence (100%). Then dithionite was added at 10 mM final concentration to quench the peptide outside the LUVs. After signal stabilization, Melittin was added (0.1 μM) to induce pores formation in the LUVs membranes and allow dithionite to quench the internalized peptide in the LUVs lumen. The residual fluorescence is due to peptide protected inside the membrane bilayer and/or between membrane aggregates. The percent of internalized peptide was calculated as the difference of fluorescence values: after dithionite minus after Melittin. For comparisons all data were normalized at 100% of the initial peptide fluorescence. A schematic representation of the protocol is shown in S1A Fig.

### Cholesterol-pyrene fluorescence

Cholesterol-pyrene fluorescence spectra were obtained with a FP 8300 Jasco fluorimeter. Emission spectra were recorded from 360 to 600 nm using a 335 nm excitation wavelength. The excitation and emission band-pass were set at 5 nm and 2.5 nm respectively. 150 μl of buffer containing 2.5 μg of LUVs were added to a quartz cell. The temperature was regulated with a Peltier device. Peptides were added at two different peptide/lipid molar ratios (P/L 1/10 and 1/25). We followed the protocol described in [38]. In brief, to analyse the cholesterol-pyrene fluorescence changes depending on temperature we used two protocols. A heating protocol which started from samples at 4°C that were heated from 10 to 55°C by 5° steps. A cooling protocol in which the samples were heated fast at 55°C and then cooled to 10°C by 5° steps. Using both protocols allows the fine characterisation and resolution of constant tendencies of peptides effects on membranes. We followed specifically the wavelengths recommended in [38]: 474 nm to characterize the cholesterol-pyrene aggregation (excimers-dimers), 373 nm which is a marker of liquid ordered (Lo) domain environment around the cholesterol-pyrene, and 379 nm which is a marker of liquid disordered (Ld) domain environment.

As described [42], fluorescence anisotropy was characterized by using the equation,

r=(Ivv-GIvh)/(Ivv+2GIvh)

in which Ivv and Ivh are the emission light intensities with the polarizers in different configurations (excitation-emission) and G is the instrumental correction factor defined as Ihv/Ihh and calculated for all wavelengths.

### Giant plasma membrane vesicles (GPMV) and fluorescence microscopy

Giant plasma membrane vesicles were obtained as described in [27] and adapted in [19]. GPMVs induction was performed by overnight incubation of cells with 1 ml of PMS buffer (1.5 mM CaCl_2_, 1.5 mM MgCl_2_, 5 mM HEPES, 1 mg ml^-1^ glucose in PBS pH 7.4). The GPMVs were extracted in 1 ml of PMS buffer, centrifuged at 1 000 rpm for 15 min in a standard Eppendorf centrifuge and recovered in 100 μl of PBS.

Microscopy observations were performed on 8 well slides (Ibidi μ-slide) containing 160 μl of freshly prepared GPMVs. For di-4-ANEPPDHQ GPMV-labelling, the probe was added at a final 5 μM concentration. Peptides were added in 10 μl for a 10 μM final concentration. Confocal images were acquired with a TCS SP2 laser-scanning spectral system (Leica) attached to a Leica DMR inverted microscope [23]. Optical sections were recorded with a 63/1.4 or 100/1.4 immersion objectives. We performed 30 confocal slices for each GPMV analysed. The samples were excited at 488 nm (Ar ion laser) and the fluorescence emission was collected at 570-590 nm for the liquid ordered (Lo) contribution and at 620-640 nm for the liquid disordered (Ld) contribution. di-4-ANEPPDHQ intensity images were converted into general polarization (GP) images with each pixel calculated in ImageJ from the two di-4-ANEPPDHQ intensity images according to the equation:

$$GP=(I_{\left(570-590\right)}-GI_{\left(620-640\right)})/(I_{\left(570-590\right)}+2GI_{\left(620-640\right)})$$

GP distributions were obtained from the GP images histogram values from regions of interests (only GPMV membranes) using a custom-build macro in ImageJ [43,44]. Three independent experiments were performed. The number of GPMVs analysed was: 32 for control GPMVs without peptides, 88 incubated with R9, 45 with RW9 and 38 with RW16.

### Statistical analyses

The graphs and the statistical treatment of experiments was performed with GraphPad Prism software. Graphs are given as means ± standard error. Unpaired, t-test were performed.

## Results

### Peptide translocation into LUVs

In order to study the translocation of CPPs through membranes that mimic the natural phospholipids of plasma membranes, we decided to use LUVs composed of eggPC (PC), and eggSM (SM) which contain natural proportions of fatty acyl chains. PC, SM/Chol and PC/SM/Chol LUVs were used to respectively mimic liquid disordered, liquid ordered and mixed liquid ordered/liquid disordered membranes.

**Fig 1:**
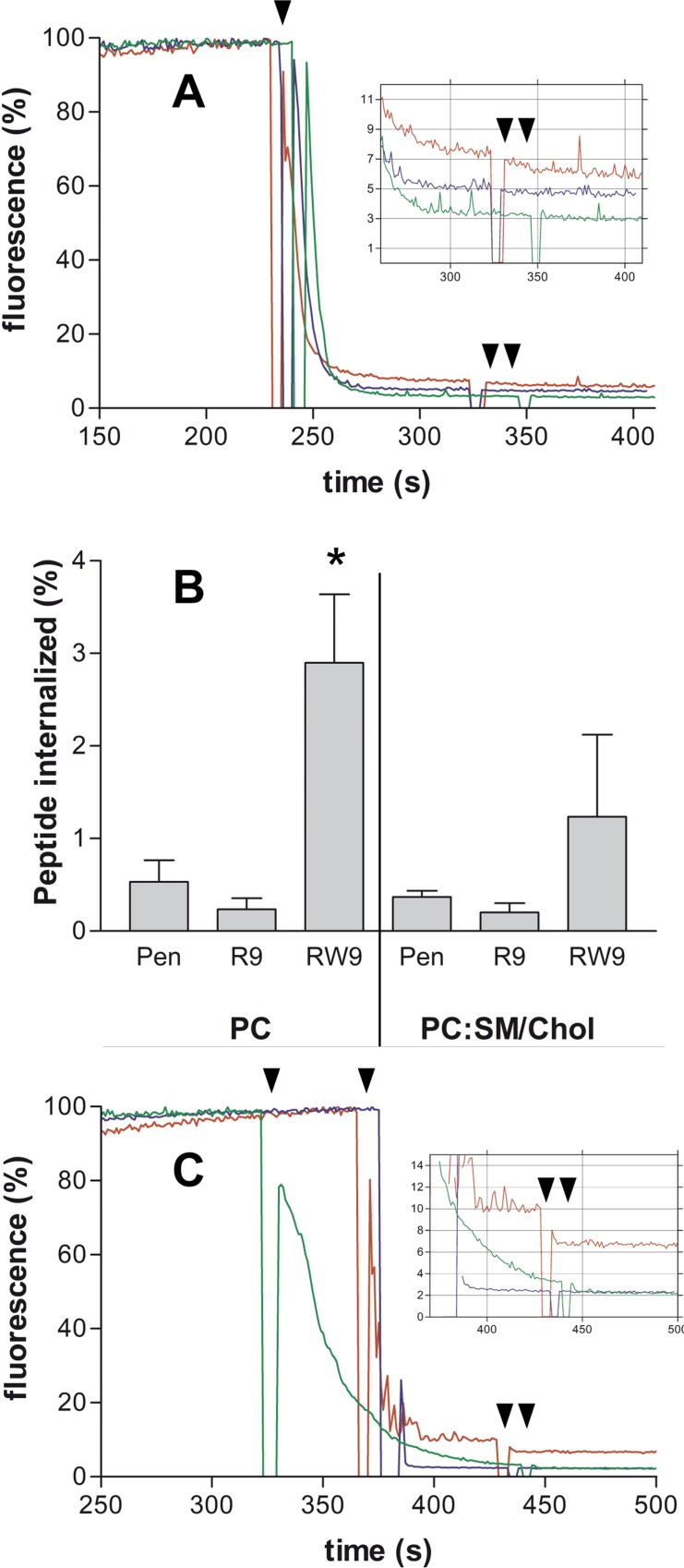
CCPs internalisation into PC LUVs. Time-dependent percent of total fluorescence evolution. (A) PC LUVs were incubated with NBD-labelled CPPs for 2.5 hours at 35°C. (B) Quantification of CPPs penetration into LUVs composed of PC and PC/SM/Chol. Means ± SEM of 3 experiments. * Indicates that the P value for RW9 was from 0.02 to 0.04 when comparing RW9 with penetratin and R9 (unpaired t-test). (C) PC LUVs incubated with NBD-labelled CPPs for 5 minutes at 50°C. In A and C dithionite was added (arrow head) to quench the CPPS in solution. After stabilization, melittin was added (two arrow heads) allowing the CPP quenching inside the LUVs. The difference of fluorescence before and after melittin is the percent of internalized peptide (Zoom). Penetratin green, R9 blue and RW9 red.

In a first series of experiments we incubated the LUVs with the peptides during 2.5 hours at 35°C. We observed that 1.5 to 4% of RW9 peptide was able to pass into the lumen of PC LUVs and occasionally in PC/SM/Chol vesicles (Fig 1A and 1B). We didn’t observe any peptide penetration for SM/Chol vesicles (S1B Fig). It has been reported that in PC and PC/SM/Chol membranes melittin permeabilizes the bilayer and that membrane permeabilization by melittin is reduced in ordered (SM/Chol) membranes [45–47]. However, in SM/Chol membranes, the intensity of peptide fluorescence after dithionite exposition was too low (background level, less than 1%) to be considered as corresponding to a quantity of internalised peptide (S1B Fig). Penetratin and R9 were unable to translocate into the LUVs lumen. The possibility that the peptides aggregate and become resistant to dithionite quenching could result in residual fluorescence. Thus, the possibility that melittin induce the dissociation of peptide aggregates and provokes a diminution of fluorescence of non LUV-internalized peptides was tested by a negative control without LUVs. The peptides were incubated in buffer, dithionite was added and, after signal stabilization, melittin was added. S2C Fig shows that melittin didn’t induce additional diminution of fluorescence indicating that the fluorescence decrease in the presence of LUVs is due to membrane permeabilization and the subsequent internalization of dithionite into LUVs and quenching of internalized peptides, and is not due to artefacts of peptides in solution.

Considering that peptide internalisation was observed with PC (Ld membranes) and not with SM/Chol (Lo membranes), we tested whether this phenomenon was correlated to the membrane fluidity performing experiments at 50°C for 5 minutes. In these conditions we observed that, RW9 crosses the PC LUVs membrane (2.5 to 3.2% of internalized peptide) (Fig 1C). No penetration was detected for SM/Chol and PC/SM/Chol. It is interesting to note that at 50°C, a temperature at which the membrane is fluid, RW9 does not penetrate into PC/SM/Chol LUVs. One explanation could be based in the fact that at high temperature the lipids are completely mixed with cholesterol distributed widely. On the contrary, at 37°C the probability of lipids separation in cholesterol-rich and poor domains increases. This suggests that cholesterol free domains or the presence of frontiers between domains are involved in peptide translocation. Overall, penetratin and R9 were unable to cross the LUVs membranes or in a very small quantity. Finally, we further incubated the LUVs and the peptides at 50°C during 45 minutes. Only RW9 was internalised into PC LUVs (5.5%). With the same experimental approach, Swiecicki and collaborators [12] had shown that, R9, RW9 and Penetratin were able to translocate into LUVs. In those experiments the membranes contained PG which increases the electrostatic affinity for these positively charged peptides. Sharmin and collaborators also showed that internalisation of R9 into GUVs required the presence of PG [33]. Herein, we used only zwitterion lipids which are representative of the extracellular leaflet of the plasma membrane. This suggests that membrane translocation of small amphipathic peptides would be less dependent on negatively charged phospholipids compared to the arginine-rich non-amphipathic peptides.

Overall, RW9 was shown to be the only peptide to cross the LUVs membranes significantly under our experimental conditions and especially to translocate through PC (liquid disordered) membranes. Therefore, to figure out why RW9 crosses the membrane, and why the presence of cholesterol reduces the internalisation into LUVs, we investigated how CPPs alters the cholesterol organization of membranes using a cholesterol-pyrene probe. Indeed, the spectral properties of the cholesterol-pyrene will inform us on (i) the formation of cholesterol enriched domains within the membrane by looking at the pyrene excimers (dimers) and on (ii) the local order of the membrane as previously characterized [38].

### Changes in cholesterol-pyrene fluorescence induced by CPPs

The changes in cholesterol-pyrene distribution and fluorescent properties induced by peptide membrane binding were studied at different temperatures using various membrane compositions. It had been shown that the lateral distribution and self-association of the probe is essentially due to the cholesterol moiety rather than to the pyrenyl group [48]. We studied cholesterol aggregation by pyrene excimer formation at 474 nm and followed the environment around the monomeric cholesterol pyrene by measuring the fluorescence intensity at 373 nm for the Lo contribution and at 379 nm for Ld contribution. As previously suggested [38], the parameters were normalized by calculating the ratios 474/432 (exci), 373/432 (cPyO3, ordered environment) and 379/432 (cPyD9, disordered environment), in which the value at 432 nm corresponds to the iso-emissive spectral point. The ratio 373/379 (cPyO3/cPyD9) was also used to evaluate the proportional changes of Lo/Ld environments (see [38]).

### Cholesterol-pyrene fluorescence in liquid disordered PC model membranes

**Fig 2:**
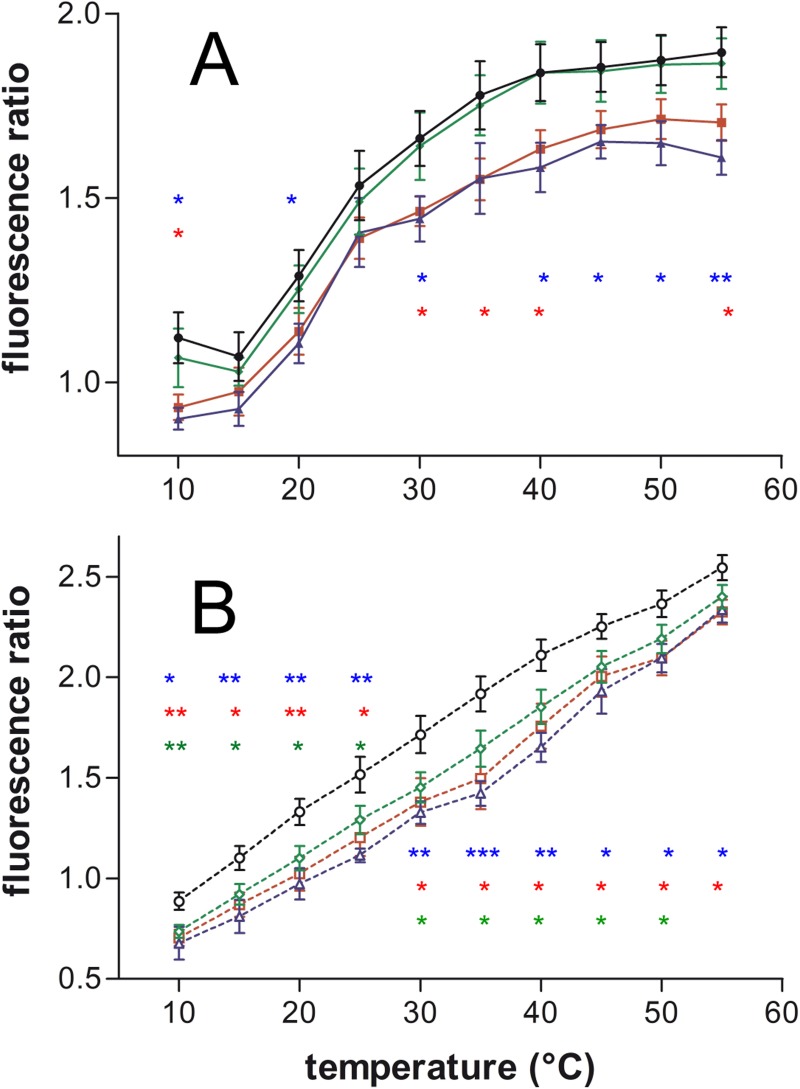
CPPs effect on cholesterol-pyrene multimers in function of temperature on PC LUVs. The excimers/isoemisive ratio (474/432 nm) was followed at different temperatures during heating (A, continuous lines), or cooling (B, dotted lines) of the samples. CPPs were incubated with the LUVs at a 1/25 P/L ratio. Control CPP free LUVs (black ●,○), Penetratin (green ♦,◊), R9 (blue ▲,∆) and RW9 (red ■,□). Means ± SEM of 5 to 8 independent experiments. The stars in colour correspond to their respective experimental point colours compared to the control LUVs; * P<0.05, ** P<0.01, *** P<0.001 by unpaired t-test.

As shown in Fig 2, at 1/25 P/L molar ratio, the three peptides provoked a diminution in the cholesterol excimer formation. With the heating protocol penetratin didn’t show any effect. On the contrary, the effects of R9 and RW9 were significant. With the cooling protocol, the peptide effect was small for penetratin, intermediate for RW9 and stronger for R9. Similar diminution in excimer formation was also observed with the peptides at 1/10 P/L ratio (S2 Fig).

**Fig 3:**
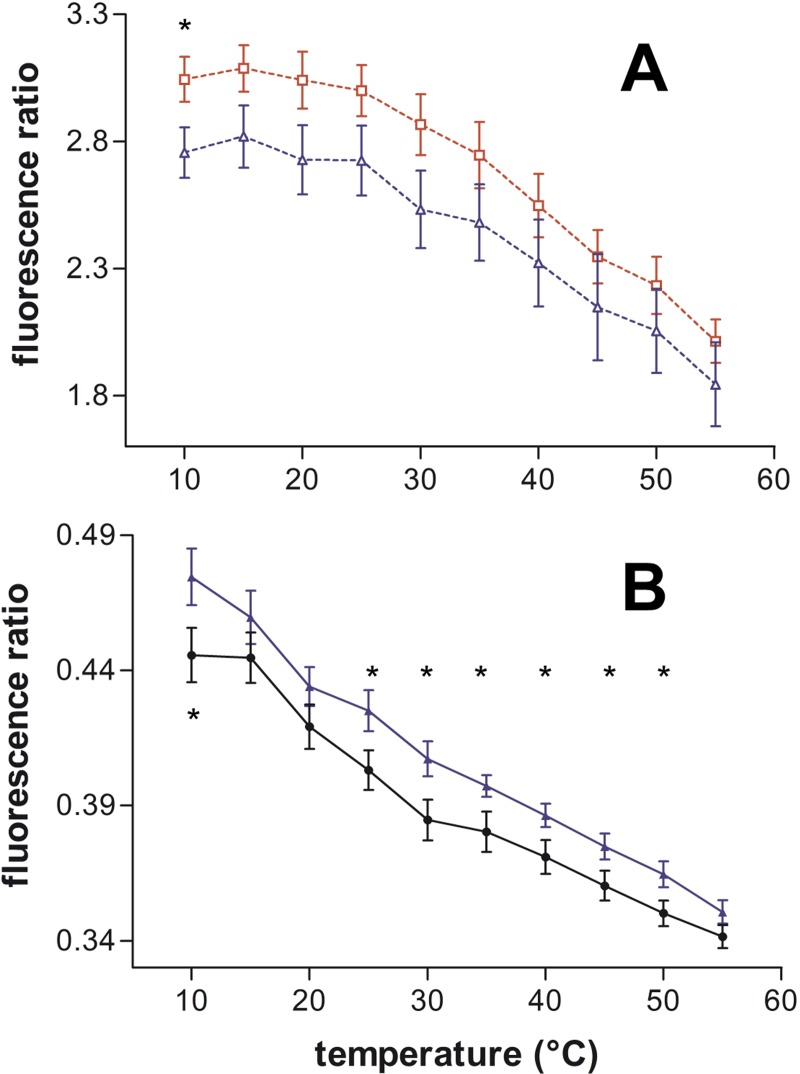
CPPs effects on the cholesterol-pyrene environment in function of temperature on PC LUVs. (A) Liquid disordered contribution (cPyD9) in PC LUVs during cooling in the presence of R9 (blue ∆) and RW9 (red □). (B) Lo/Ld ratio (cPyO3/cPyD9) in PC LUVs during heating in the absence (black ●) or the presence of R9 (blue ▲) at P/L ratio 1/25. Means ± SEM of 5 to 7 independent experiments. * P<0.05 by unpaired t-test.

The Ld (cPyD9) contribution was not modified by the peptides during membrane heating (S3A and S3C Fig). However, with the cooling protocol, we observed that at P/L ratio 1/25, R9 showed a small tendency to decrease the Ld contribution, especially at low temperatures, and that RW9 increased slightly the Ld contribution at high temperatures (S3B Fig). At 1/10 P/L ratio the effect was only observed at low temperatures (S3D Fig). Fig 3A shows the difference in cPyD9 values between R9 and RW9 (1/25 P/L). Penetratin did not change the Ld contribution.

In agreement with the presence of about 45% of saturated acyl chains in eggPC, the cholesterol-pyrene detected an Lo environment due to its association with saturated chains [30]. Concerning this Lo contribution (cPyO3), R9 and RW9 showed a small increase in the cPyO3 value, especially with the heating protocol. Penetratin did not show any effect (S3E–S3H Fig). The analyses of the cPyO3/cPyD9 (Lo/Ld) ratio, which is more accurate parameter to measure membrane order changes than the cPyO3 and cPyD9 [38], showed that penetratin had the smaller effect and that R9 and RW9 increased the Lo/Ld ratio (S3I–S3L Fig). This increase in Lo character was already present for R9, especially when applying the “heating protocol” at P/L 1/25, as shown in Fig 3B.

### Cholesterol-pyrene changes in liquid ordered model membranes

**Fig 4:**
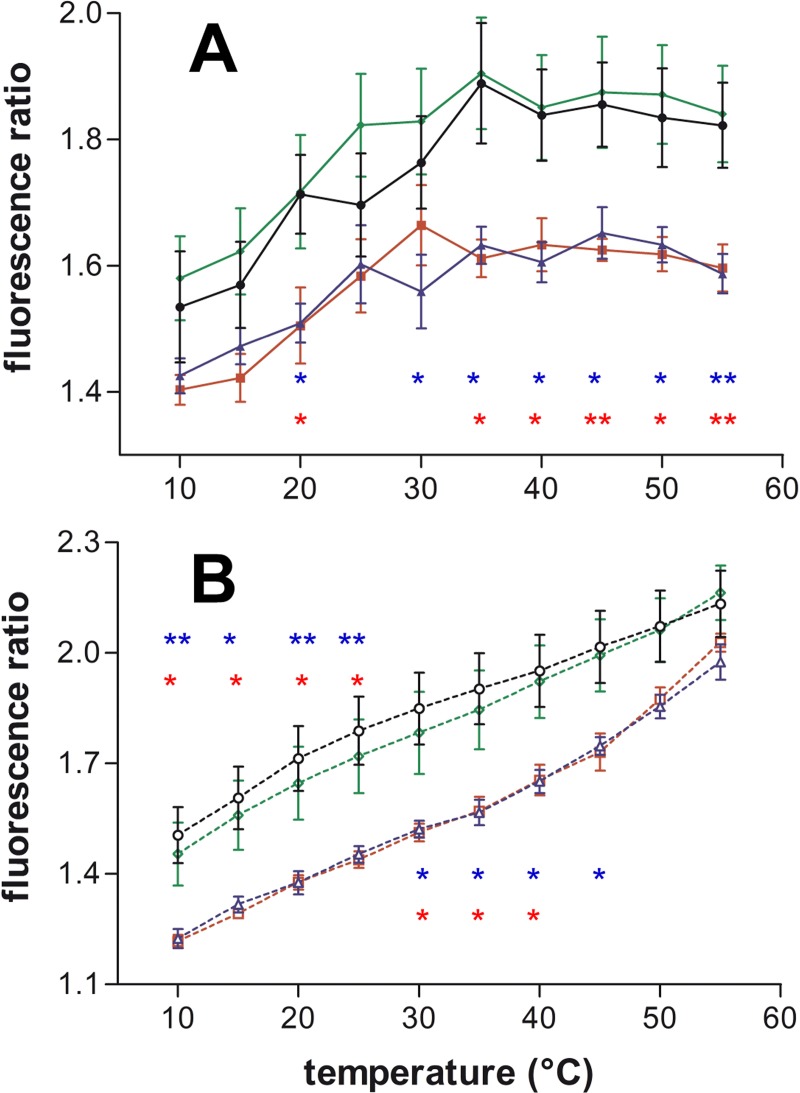
CPPs effect on cholesterol-pyrene multimers in function of temperature on SM/Chol LUVs. The excimers/iso-emissive ratio (474/432 nm) was followed during heating (A, continuous lines), or cooling (B, dotted lines). CPPs were incubated with the LUVs at 1/10 P/L ratio. Control CPP free LUVs (black ●,○), Penetratin (green ♦,◊), R9 (blue ▲,∆) and RW9 (red ■,□). Means ± SEM of 3 to 9 independent experiments. The stars in colour correspond to their respective experimental point colours compared to the control LUVs; * P<0.05, ** P<0.01 by unpaired t-test.

The experiments with SM/Chol membranes showed that the excimer formation (cholesterol aggregation) was not modified by penetratin. On the contrary, R9 and RW9 showed a decrease in excimers (Fig 4, 1/10 P/L and S4 Fig, 1/25 P/L).

The Ld contribution was not perturbed with penetratin but the other peptides showed an increase in Ld character of the cholesterol-pyrene environment (S5 Fig). The effects on the Lo contribution were too small to be considered. The changes in the Lo/Ld ratio showed also a very small effect with the heating protocol, a small decrease for R9 and more important for RW9 during cooling.

### Cholesterol-pyrene changes in heterogeneous membranes

The changes in excimers formation in PC/SM/Chol membranes were similar to those observed in PC membranes. Overall, penetratin show no significant decrease in excimer contribution, RW9 show and intermediate effect and the strongest excimers reduction was observed with R9 (S6 Fig).

The R9 and RW9 peptides induce an increase in the Ld contribution specially during the cooling protocol (S7 Fig) and the effect on the Lo contribution was very small, as it was the case for SM/Chol membranes. In these heterogeneous membranes, the peptide effects were different depending on the P/L ratio. At 1/10 P/L, peptides effects resembled to those observed with SM/Chol membranes. At 1/25 P/L, peptides effects were similar to the behaviour of PC membranes (compare S3, S5 and S7 Figs). This suggests that in these heterogeneous membranes, PC enriched domains are able to be modified at low peptide concentrations whereas SM/Chol domains are sensitive only at high peptide concentration.

Table 1 summarizes the peptides effects on the cholesterol redistribution in different membranes. Briefly, penetratin induced small changes on cholesterol distribution. R9 induced the stronger reduction in excimers. Accordingly, R9 increases strongly the Lo/Ld ratio in PC and RW9 only slightly. In SM/Chol membranes the changes in fluidity were small. Finally, in mixed membranes (PC/SM/Chol), at low concentrations the peptides showed similar effects as in PC and at high concentration to SM/Chol membranes.

**Table 1:** Peptide effects on cholesterol-pyrene fluorescence ratios in different membranes.

Membrane	Peptide	cholesterol aggregation exci/iso (*a*)	membrane order ratio cPyO3/cPyD9 (*a*,*b*)
	R9	- - -	**+ + +**
PC	RW9	- -	**+**
	Penetratin	-	**+ +**
	R9	- - -	-
SM/Chol (1/1)	RW9	- - -	-
	Penetratin	n	n
	R9	- - -	LC **++** HC - -
PC/SM/Chol (1/1/1)	RW9	- -	LC **+** HC -
	Penetratin	-	LC **+** HC n

*a*; the sign - indicates a decrease and **+** an increase in the corresponding ratios values, n: no effect.

*b*; LC indicates membrane effects at low peptide concentration (P/L 1/25), and HC at high peptide concentration (P/L 1/10).

### Cholesterol-pyrene movement amplitude by anisotropy

**Fig 5:**
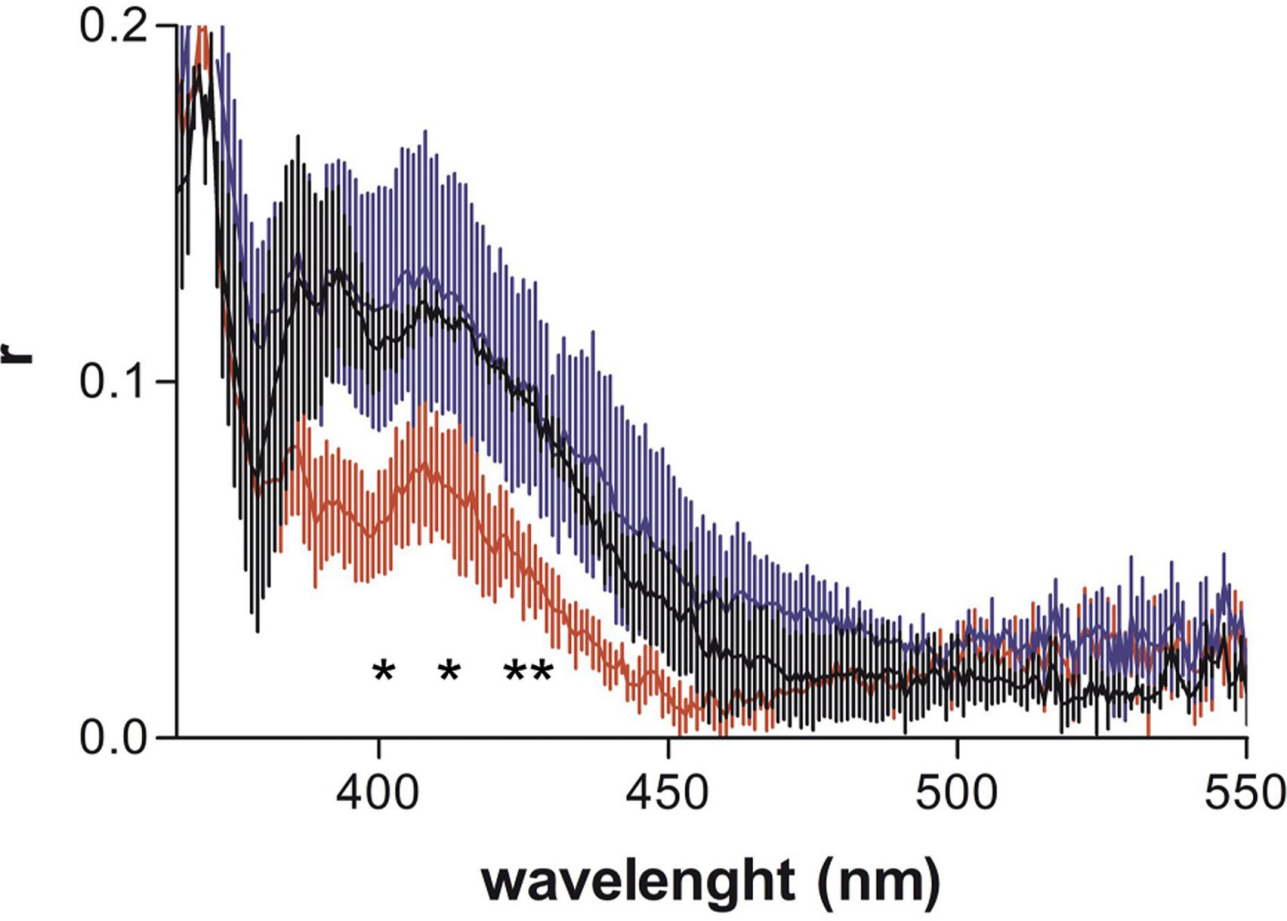
CPPs effect on cholesterol-pyrene fluorescence anisotropy. SM/Chol membranes were incubated at 35°C with R9 in blue or RW9 in red. Control peptide free LUVs are in Black. Means ± SEM of 3 independent experiments. Unpaired t-test was performed for 390, 400, 410 and 420 nm. The stars correspond to the comparison of RW9 and control LUVs; * P<0.05, ** P<0.01.

To characterize the movement amplitude of the cholesterol-pyrene in the absence and presence of the peptides at 1/10 P/L ratio, we measured pyrene fluorescence anisotropy. We observed that at 15°C and 50°C the membranes are either too rigid or fluid to observe significant differences in the presence of peptides. However, at 35°C for SM/Chol membranes, we observed that RW9 induces a significant decrease in anisotropy in the range of 390-430 nm, which corresponds to the contribution of monomeric cholesterol-pyrene (Fig 5). On the contrary, peptides showed no effect on the contribution of the cholesterol-pyrene multimeric form (430-550 nm range). In other words, in SM/Chol membranes, RW9 favours the movement of monomeric cholesterol-pyrene. In PC membranes we also observed a small decrease in anisotropy of monomeric cholesterol-pyrene in the presence of RW9 and a slight increase in anisotropy of the multimeric form with R9. Penetratin showed no changes in agreement with previous data describing no major changes in PC/PG (4/1) bilayer structure [49].

### CPPs effects on giant plasma membrane vesicles (GPMVs) ordering

The cholesterol-pyrene probe studies suggest that RW9 increases the fluidity of the membrane of LUVs (Ld character). Therefore, in order to study peptides effects on cellular membranes order, we performed experiments on plasma membrane giant vesicles from MDCK cells labelled with the environmental probe di-4-ANEPPDHQ. This probe, similarly to Laurdan, have been used to characterize membrane domains [50–52]. Recently, Amaro and collaborators [53] showed that compared to Laurdan, this probe is less sensitive to lipid packing and polarity, but it is more sensitive to the cholesterol and dielectric constant of the environment. This fluorophore can consequently be used to probe the cholesterol enriched (Lo) domains. As cholesterol increases membrane order, we use the term “ordering” to discuss the spectral changes of the probe.

**Fig 6:**
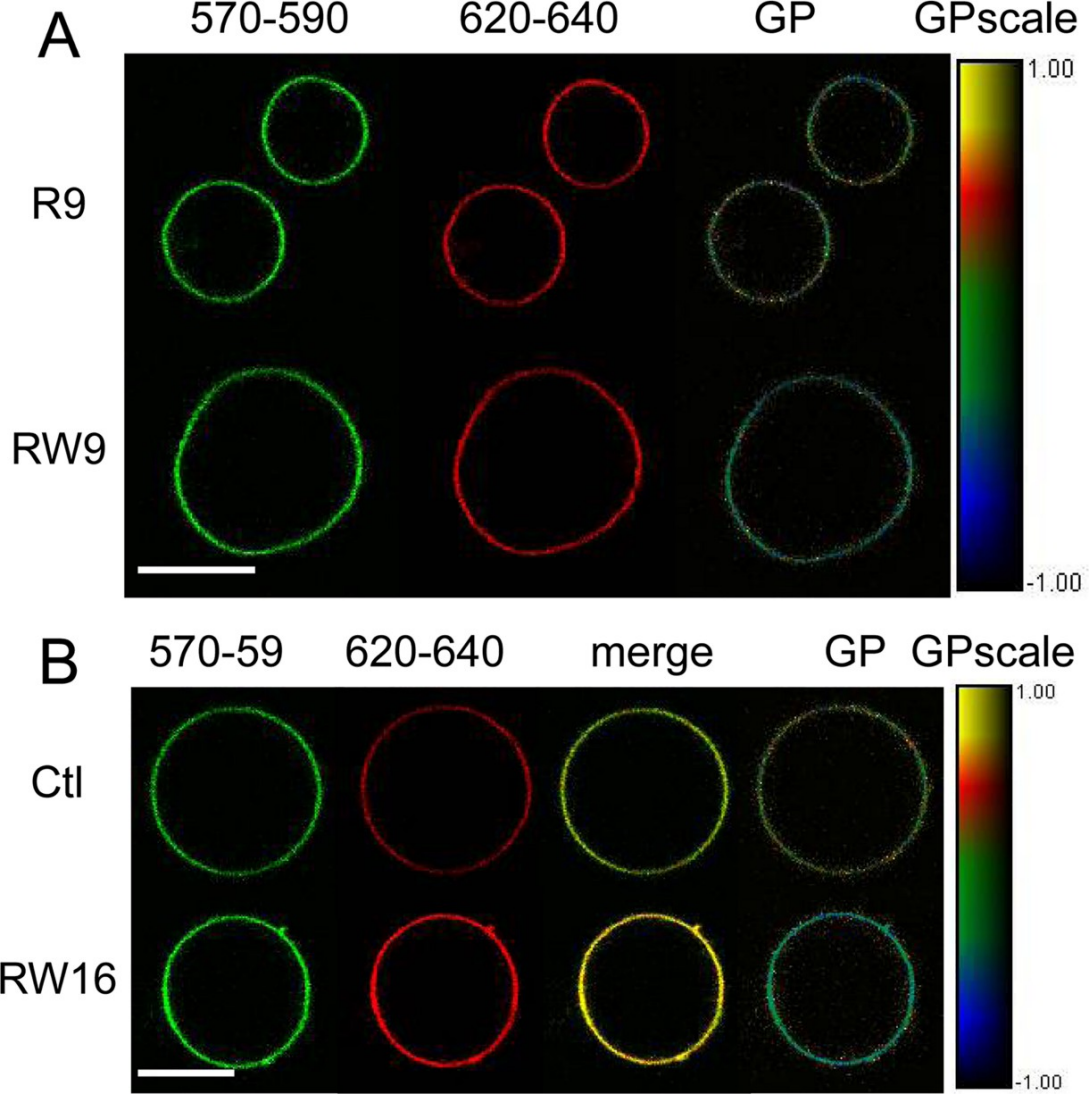
Confocal images of GPMVs labelled with di-4-ANEPPDHQ and incubated with CPPs. The 570-590 nm range corresponds to the ordered membrane contribution and the 620-640 nm range to the disordered membrane contribution. GP was calculated as explained in the methods section. (A) GPMVs incubated with R9 (top) and RW9 (bottom) during 190 min. (B) Peptide-free GPMV (top) and incubated with RW16 (bottom) during 60 min. Bars represent 10 μm.

**Fig 7:**
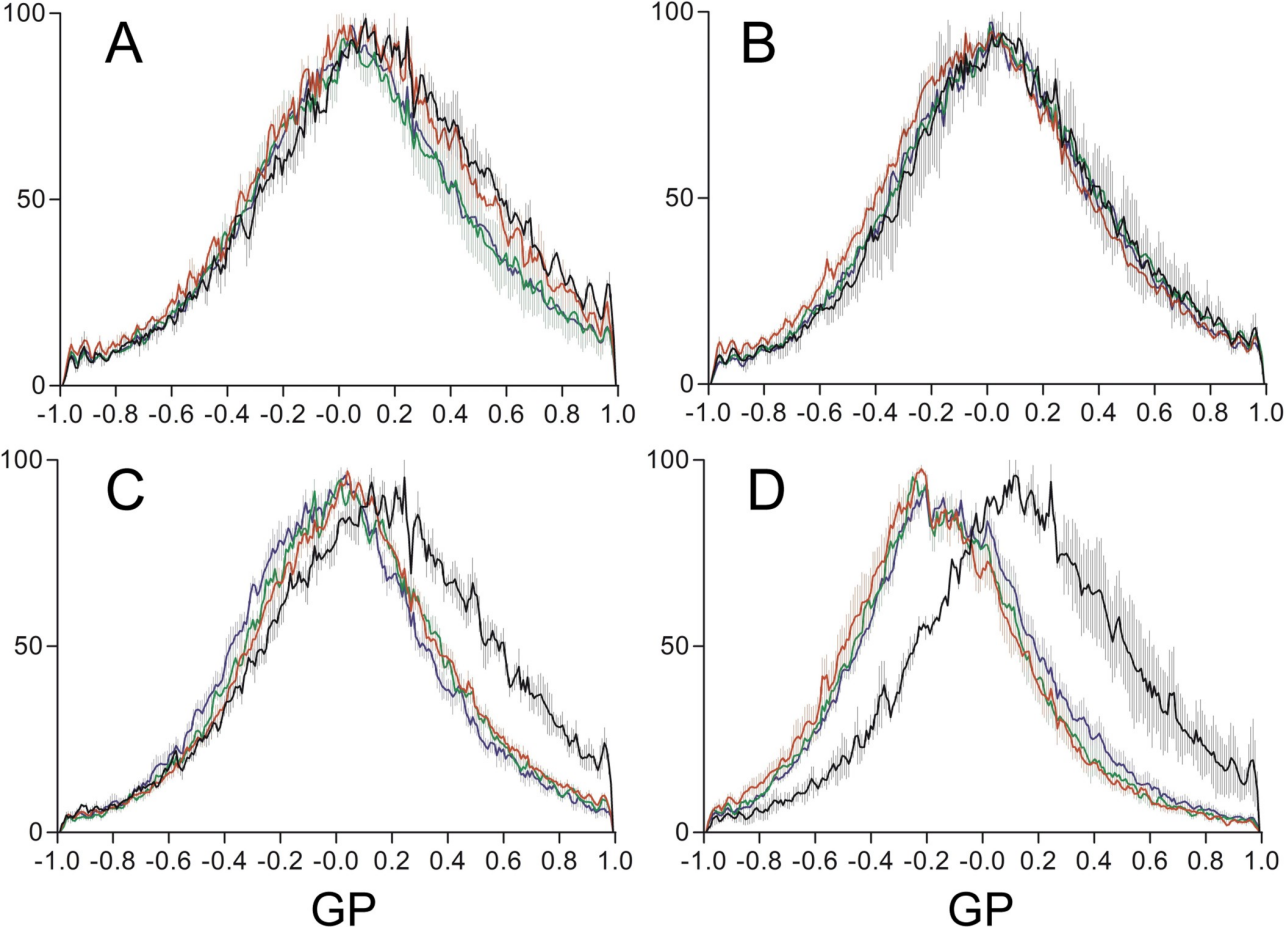
Time evolution of GP histograms of di-4-ANEPPDHQ-labelled PMS incubated with CPPs. Pixel GP distribution of PMS incubated without peptide (A), with R9 (B), RW9 (C) and RW16 (D). The incubation times with or without peptides are 5-20 min in black, 20-90 min in red 90-140 min green and 140-200 min in blue. Distributions are means ± SEM of 3 to 10 GPMVs.

We incubated the GPMVs together with CPPs and followed the changes in membrane lipid ordering by measuring the evolution of the GP parameter during 200 minutes. Penetratin induced only small changes of cholesterol-pyrene fluorescence in the LUVs, so we focused on the study of R9 and RW9, two peptides of the same size but displaying different behaviour for LUV internalisation and cholesterol-pyrene fluorescence changes. Fig 6A shows confocal images of GPMVs incubated with R9 and RW9 and the corresponding GP images. The GP images show clearly that for the GPMVs incubated with RW9, the pixels colours were shifted to the blue compared to the GPMVs incubated with R9. Fig 7 shows the quantitative data of GP pixel distribution for different peptides. The mean GP value showed fluctuations during the time of the experiment but is relatively stable. Compared to the GPMVs incubated in the absence of peptides, R9 only induced minor changes in the mean GP value (Fig 7B). On the contrary, RW9 induced a shift towards the negative GP values (0.1-0.2 GP units, Fig 7C). This clearly indicates that RW9 increases membrane lipid disordering.

R9 and RW9 peptides differ by the presence of tryptophan residues and by the propensity of RW9 to adopt an amphipathic alpha helical structure. Therefore, we tested the role of amphipathicity on membrane fluidity induction by using the 16 residues peptide RW16 which contains 6 tryptophan residues. Fig 6B shows that RW16 induces a clear change in the GP value as seen by the strong blue shit of the image. Moreover, the effect is clear in the merged image in which the GPMV incubated with RW16 is yellow compared to the green-shifted control GPMV. This strong effect was also observed on the GP pixel distributions (Fig 7). RW16 induced a stronger shift in the GP values (0.1-0.3 GP units). These data suggest that the presence of the tryptophan residues and the propensity to acquire α-helical conformation are involved in membrane disorder induction.

## Discussion

The purpose of this work was to study the role of lipid organisation during CPP-membrane interaction. We performed experiments with membrane containing phospholipid from natural sources mimicking biological membranes which contain acyl chains of different length and degree of unsaturation. Secondly, we studied the peptides effects on lipid order in giant plasma membrane vesicles obtained from epithelial cells.

The peptide translocation experiments showed that, under our experimental conditions, RW9 was able to cross the membrane of LUVs containing liquid disordered domains but not in LUVs in which only the liquid ordered domain (SM/Chol) was present. Penetratin and R9 were unable to cross any of the membranes that we tested or in a small quantity, below the experimental protocol threshold sensitivity. These results also indicate that the membranes used in this study are selective, and that membrane fluidity allows the translocation of RW9. It has been shown that different arginine-rich CPPs are able to cross membranes within different efficacies [5,54]. However, in the present conditions, only RW9 was internalized efficiently. The most significant difference between R9 and RW9 is that RW9 contains three tryptophan residues which allows the formation of an amphipathic α-helix. It is important to notice that both, peptide secondary structure and the increase in hydrophobicity have been considered to play fundamental roles for membrane translocation [55–57]. Moreover, comparative studies with membrane monolayers showed that RW9 is able to insert into POPC and DPPC monolayers but that the insertion of R9 was absent or very small [58,59]. The membrane insertion (snorkelling [60]) of a tryptophan-containing amphipathic α-helix could increase membrane fluidity. Taking together, these new data suggest that tryptophans and the formation of an α-helix could be important to increase membrane fluidity and enhance membrane translocation.

We observed that the cholesterol-pyrene excimers was reduced upon peptide-membrane interaction. As previously discussed [38], the reduction of cholesterol excimers is due in part to the redistribution of lipids and in part to a decrease in global fluidity of the membrane which reduces the thermal molecular collisions. However, the peptide-induced reduction of excimers is not necessarily accompanied by a reduction in membrane fluidity (RW9 for example). This indicates that a true peptide-induced lipid reorganisation takes place. The reduction in excimers is necessarily accompanied by an increase in the number of monomeric cholesterol-pyrene molecules. This phenomenon was small for penetratin and pronounced for R9 and RW9. The peptide-induced increase in the distance between cholesterol-pyrene molecules can be interpreted in two different ways.

**Fig 8:**
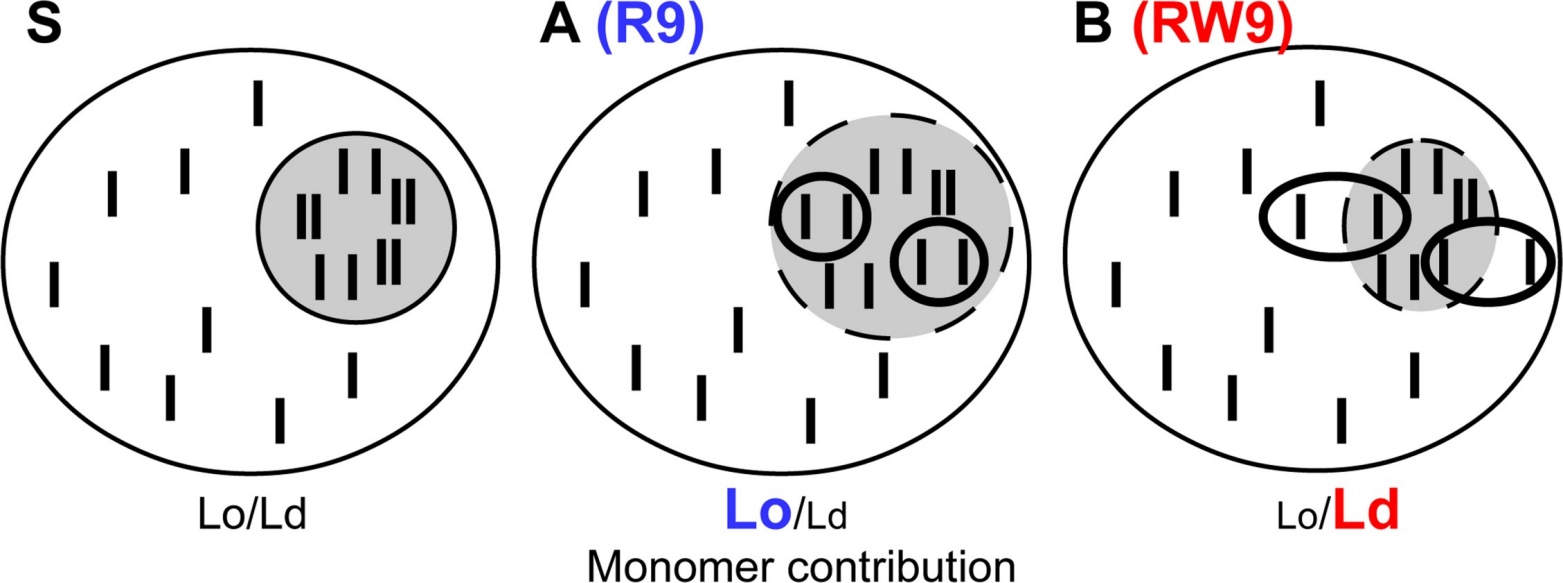
Schematic representation of cholesterol-pyrene movements in the membrane. A cell membrane is composed of different domains of disordered (white) and ordered (grey) character. In a starting condition (S), the cholesterol-pyrene probe (black bars) is distributed in all domains but enriched in Lo domains in which it is frequently present as dimers (multimers). If cholesterol-pyrene dimers dissociate inside the Lo domain (A), the new monomeric species would increase the Lo spectral signal contribution and consequently the Lo/Ld ratio. If the cholesterol-pyrene newly formed monomers move to an Ld domain the Ld spectral contribution increases and the Lo/Ld ratio diminishes (B). The presented experimental data suggest that R9 effect on PC membranes is well represented by the A hypothesis and that the cholesterol reorganisation induced by RW9 rather follows the hypothesis B.

First (hypothesis A, Fig 8A), the cholesterol enriched domains contribution could increase and the probe would be diluted in this Lo domains. In this case, the Lo fluorescence contribution due to newly formed monomers increases resulting in higher Lo/Ld ratios.

Second (hypothesis B, Fig 8B), the cholesterol-pyrene could migrate from an Lo domain and reach an Ld domain. In this case, the Lo/Ld ratio must diminish if all the new monomers move to an Ld domain.

These hypotheses consider that all the monomers stay in the Lo domains (A), or move to the Ld domains (B). However, it is quite likely that some proportion of monomers escape the Lo domains and other proportion remains in the Lo domain. As a consequence, the Lo/Ld ratio could rise, decrease or remain stable. In all cases, the contribution of the cholesterol-enriched membrane domains change.

The comparison of the peptide-induced modifications on cholesterol-pyrene fluorescence, revealed different behaviour depending on the peptide and the membrane (Table 1). In PC membranes R9 provoked a strong increase in Lo/Ld ratio, in agreement with hypothesis A. On the contrary, RW9 showed a lower increase in the Lo/Ld ratio but an increase in the Ld contribution and a decrease in anisotropy which corresponds better with the hypothesis B. For SM/Chol membranes the excimer reduction was stronger for R9 and RW9 compared to penetratin however, the Lo/Ld ratio decreased. This is in disagreement with the first hypothesis (A) which imply an increase in Lo contribution. However, it is coherent with the fact that the order of an already quite ordered domain (SM/Chol) could not be highly increased. Concerning PC/SM/Chol membranes, the peptides showed a more complex behaviour. At low peptide concentration (1/25), the Lo/Ld behaviour was similar to what is observed with PC and at higher concentration (1/10), to the SM/Chol. This suggests that at low concentration these CPPs are able to modify fluid (Ld) membranes and in the other hand, a higher concentration is required to modify the Lo membranes.

Our experiments with GPMVs showed that R9 did not modify significantly the membrane fluidity. On the contrary, and in agreement with experiments carried out with model membranes [20], RW9 was able to increase membrane disorder moderately. Moreover, RW16 which forms a longer amphipathic alpha helix, induces a stronger increase in GPMVs disordering. This is in agreement with experiments suggesting that lipid packing loosening promotes the oligo-arginine peptides entry into cells [61].

Depending on peptide concentration, RW9 showed different behaviour suggesting two different modes of action; one driven by electrostatic interaction which involves arginine residues and a second involving the hydrophobic tryptophan residues. For example, at high peptide concentration, the effects of R9 and RW9 on membranes are similar suggesting a common mechanism of membrane interaction by the arginine residues (S3K Fig). On the contrary, at low peptide concentration peptides behave differently (S3I Fig), indicating a different binding mode in which RW9 would bind to the membrane by the hydrophobic moiety (the tryptophan-rich side of the amphipathic helix). These possibilities are supported by simulations suggesting that the amphipathic peptides are able to interact with the membrane by the arginine residues and change orientation with insertion of the hydrophobic residues into the membrane [62].

## Conclusion

In conclusion, penetratin induced small modifications in the cholesterol-pyrene distribution and did not cross the LUVs membranes. R9 triggered a diminution in cholesterol-pyrene aggregation and a slight increase in the Lo membrane environment contribution. Therefore, R9 modifies the membranes following the cholesterol movements illustrated in Fig 8A. Similarly, RW9 reduced the cholesterol-pyrene aggregation. However, RW9 favours the Ld environment of cholesterol-pyrene. This behaviour is represented in the scheme of Fig 8B. Moreover, the propensity of RW9 to favour the Ld domains was supported by its effect on membrane lipid de-packing observed in GPMVs. Overall, our data suggest that the membrane cholesterol is redistributed during peptide-membrane interaction, that the peptide-induced disorder favours the translocation of RW9, and that the peptide amphipathicity is important to induce both, membrane fluidity and peptide translocation through the membrane. We suggest that in cells, the peptides are able to induce lipid reorganization including cholesterol movements. This lipid rearrangements result in the preferential peptide translocation through the plasma membrane in liquid disordered (non-raft) domains or at the interface of ordered-disordered domains.

## Supporting information

S1 Fig CCPs internalisation into LUVs. (A) Protocol for time-dependent percent of total fluorescence evolution. LUVs were incubated with NBD-labelled CPPs. Dithionite is added to quench free in solution CPPs. After stabilization, melittin was added allowing the quenching of CPP inside the LUVs lumen. Therefore, the difference of fluorescence before and after melittin is the internalized peptide in percent. 100% is the initial peptide fluorescence. (B) SM/Chol LUVs were incubated with NBD-labelled CPPs for 2.5 hours at 35°C. (C) NBD-labelled CPPs were incubated at 35°C in the absence of LUVs. In A and C dithionite was added (arrow) to quench the CPPS in solution. After stabilization, melittin was added (two arrows and zoom panels) allowing the CPP quenching inside the LUVs in B. Penetratin black, R9 green and RW9 red.
(JPG)

S2 Fig CPPs effect on cholesterol-pyrene multimers in function of temperature on PC LUVs. The excimer/iso-emissive ratio (474/432 nm) was followed at different temperatures during heating (A), or cooling (B). CPPs were incubated with the LUVs at a 1/10 P/L ratio. Control CPP free LUVs (black ●,○), Penetratin (green ♦,◊), R9 (blue ▲,∆) and RW9 (red ■,□). Means ± SEM of 5 to 7 independent experiments. The stars in colour correspond to their respective experimental point colours compared to the control LUVs; * P<0.05, ** P<0.01 by unpaired t-test.
(JPG)

S3 Fig CPPs effects on cholesterol-pyrene fluorescence parameters (cholesterol environment) in function of temperature on PC LUVs. The different ratios were followed at different temperatures during heating (continuous lines), or cooling (dotted lines) of the samples. CPPs were incubated with the LUVs at a 1/25 P/L ratio (top panels) and 1/10 ratio (bottom panels). The Liquid disordered contribution (Ld) is quantified by the 379/432 nm ratio (cPyD9). The Liquid ordered contribution (Lo) is quantified by the 373/432 nm ratio (cPyO3). The balance of Lo/Ld contributions by the 373/379 ratio (cPyO3/cPyD9). Control CPP free LUVs black, Penetratin green, R9 blue and RW9 red. Means ± SEM of 5 to 8 independent experiments.
(JPG)

S4 Fig CPPs effect on cholesterol-pyrene multimers in function of temperature on SM/Chol LUVs. The excimers/isoemisive ratio (474/432 nm) was followed at different temperatures during heating (A), or cooling (B) of the samples. CPPs were incubated with the LUVs at a 1/25 P/L ratio. Control CPP free LUVs black, Penetratin green, R9 blue and RW9 red. Means ± SEM of 4 to 8 independent experiments. The stars in colour correspond to their respective experimental point colours compared to the control LUVs; * P<0.05, ** P<0.01 by unpaired t-test.
(JPG)

S5 Fig CPPs effects on cholesterol-pyrene fluorescence liquid disordered environment in function of temperature on SM/Chol LUVs. The 379/432 ratios were followed at different temperatures during heating (continuous lines), or cooling (dotted lines) of the samples. CPPs were incubated with the LUVs at a 1/25 P/L ratio (top panels) and 1/10 ratio (bottom panels). The Liquid disordered contribution (Ld) is quantified by the 379/432 nm ratio (cPyD9). Control CPP free LUVs black, Penetratin green, R9 blue and RW9 red. Means ± SEM of 4 to 9 independent experiments.
(JPG)

S6 Fig CPPs effect on cholesterol-pyrene multimers in function of temperature on PC/SM/Chol LUVs. The excimers/isoemisive ratio (474/432 nm) was followed at different temperatures during heating (A), or cooling (B) of the samples. CPPs were incubated with the LUVs at a 1/25 P/L ratio. At a 1/10 P/L ratio the peptides effects on excimers were identical to those at 1/25 ratio. Control CPP free LUVs black, Penetratin green, R9 blue and RW9 red. Means ± SEM of 5 to 8 independent experiments. The stars in colour correspond to their respective experimental point colours compared to the control LUVs; * P<0.05, ** P<0.01, *** P<0.001 by unpaired t-test.
(JPG)

S7 Fig CPPs effects on cholesterol-pyrene liquid disordered environment in function of temperature on PC/SM/Chol LUVs. The different ratios were followed at 379/432 temperatures during heating (continuous lines), or cooling (dotted lines) of the samples. CPPs were incubated with the LUVs at a 1/25 P/L ratio (top panels) and 1/10 ratio (bottom panels). The Liquid disordered contribution (Ld) is quantified by the 379/432 nm ratio (cPyD9). Control CPP free LUVs black, Penetratin green, R9 blue and RW9 red. Means ± SEM of 5 to 8 independent experiments.
(JPG)
