# Supplementary figures and images for: Cholesterol re-organisation and lipid de-packing by arginine-rich cell penetrating peptides: Role in membrane translocation
Source: PLoS One. 2019 Jan 23;14(1):e0210985. doi: 10.1371/journal.pone.0210985 (PMC6343925; doi:10.1371/journal.pone.0210985)

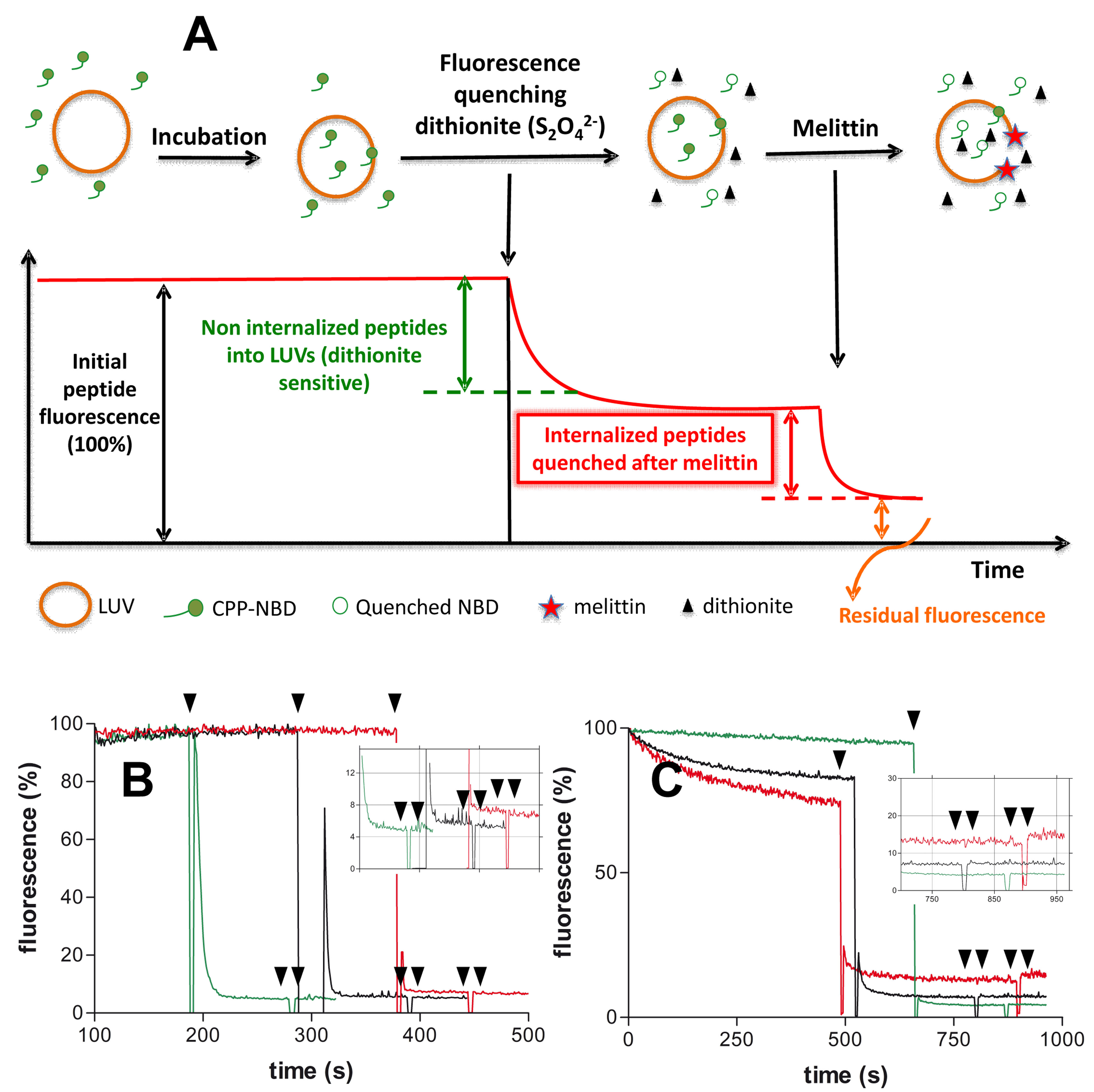

Supplement: S1 Fig — (A) Protocol for time-dependent percent of total fluorescence evolution. LUVs were incubated with NBD-labelled CPPs. Dithionite is added to quench free in solution CPPs. After stabilization, melittin was added allowing the quenching of CPP inside the LUVs lumen. Therefore, the difference of fluorescence before and after melittin is the internalized peptide in percent. 100% is the initial peptide fluorescence. (B) SM/Chol LUVs were incubated with NBD-labelled CPPs for 2.5 hours at 35°C. (C) NBD-labelled CPPs were incubated at 35°C in the absence of LUVs. In A and C dithionite was added (arrow) to quench the CPPS in solution. After stabilization, melittin was added (two arrows and zoom panels) allowing the CPP quenching inside the LUVs in B. Penetratin black, R9 green and RW9 red. (JPG) [file pone.0210985.s001.jpg]

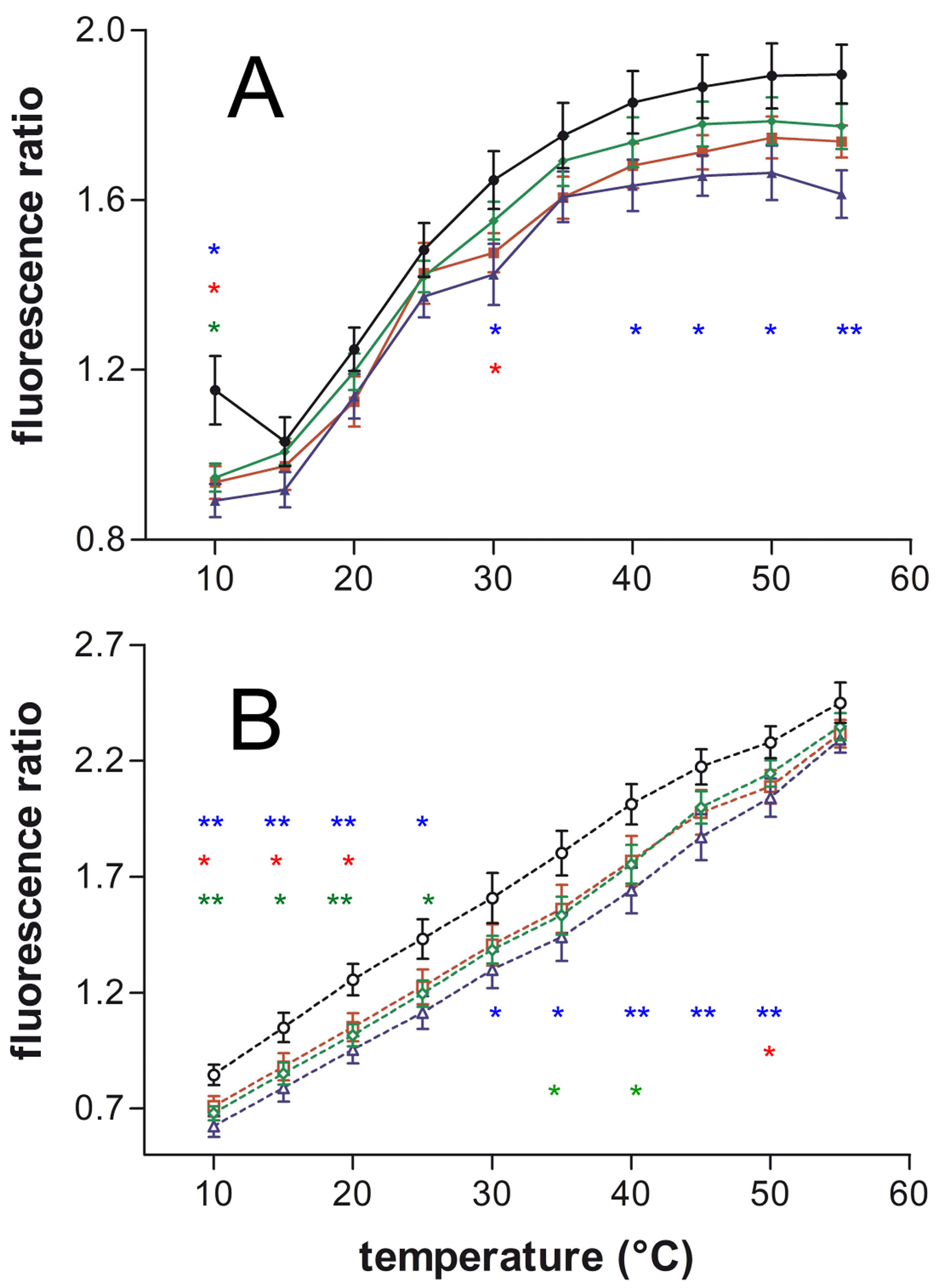

Supplement: S2 Fig — The excimer/iso-emissive ratio (474/432 nm) was followed at different temperatures during heating (A), or cooling (B). CPPs were incubated with the LUVs at a 1/10 P/L ratio. Control CPP free LUVs (black ●,○), Penetratin (green ♦,◊), R9 (blue ▲,∆) and RW9 (red ■,□). Means ± SEM of 5 to 7 independent experiments. The stars in colour correspond to their respective experimental point colours compared to the control LUVs; * P<0.05, ** P<0.01 by unpaired t-test. (JPG) [file pone.0210985.s002.jpg]

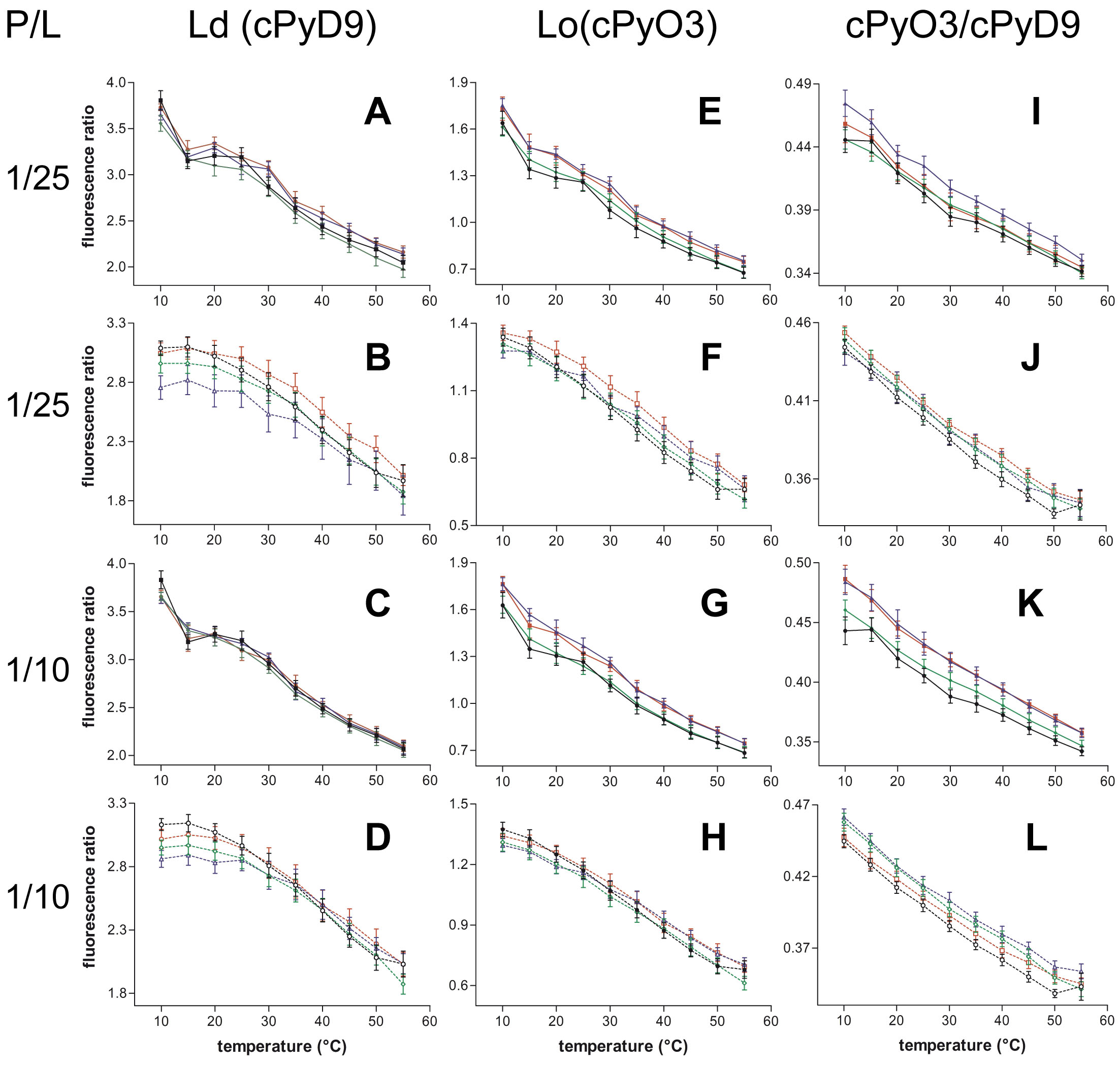

Supplement: S3 Fig — The different ratios were followed at different temperatures during heating (continuous lines), or cooling (dotted lines) of the samples. CPPs were incubated with the LUVs at a 1/25 P/L ratio (top panels) and 1/10 ratio (bottom panels). The Liquid disordered contribution (Ld) is quantified by the 379/432 nm ratio (cPyD9). The Liquid ordered contribution (Lo) is quantified by the 373/432 nm ratio (cPyO3). The balance of Lo/Ld contributions by the 373/379 ratio (cPyO3/cPyD9). Control CPP free LUVs black, Penetratin green, R9 blue and RW9 red. Means ± SEM of 5 to 8 independent experiments. (JPG) [file pone.0210985.s003.jpg]

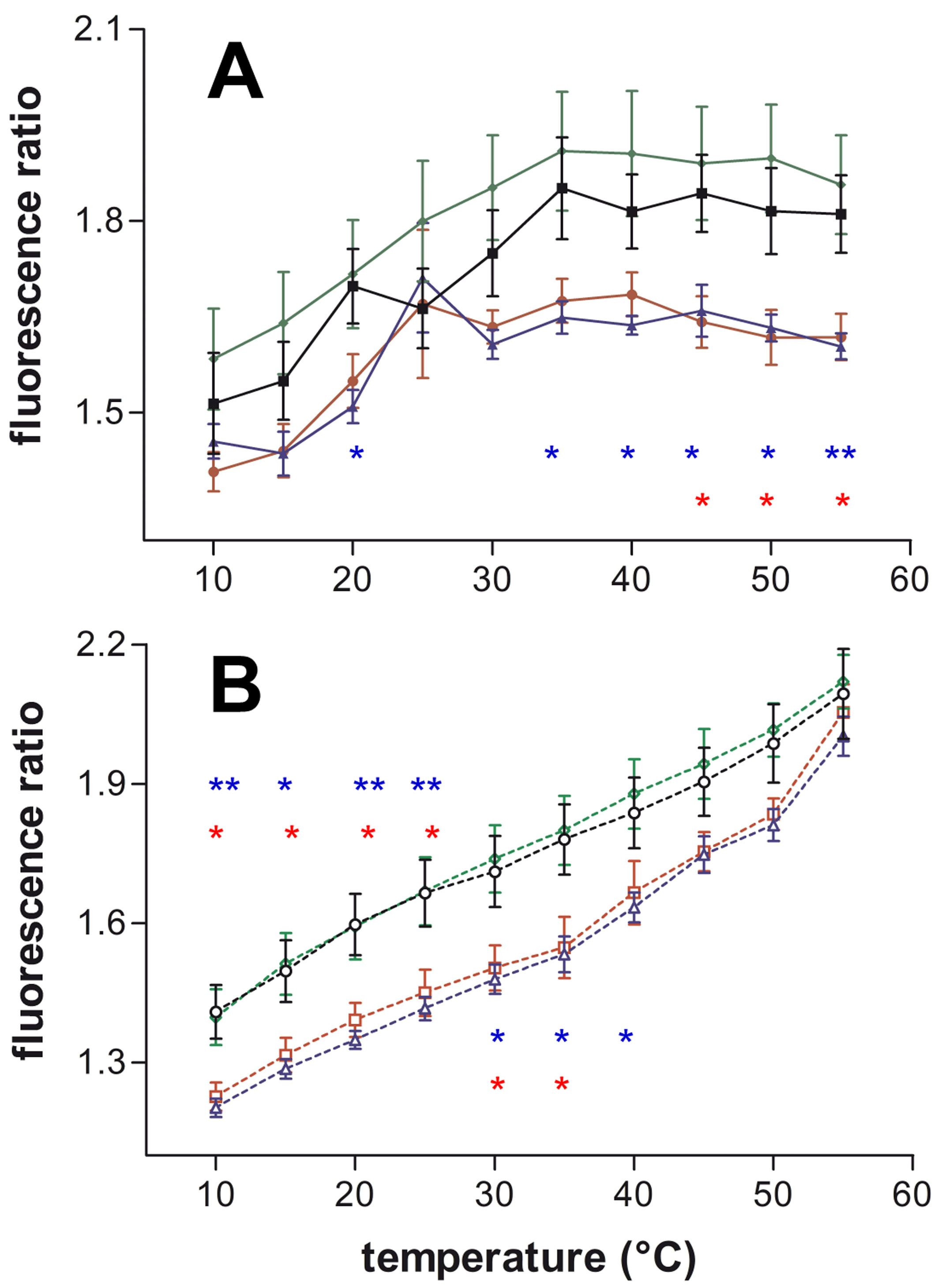

Supplement: S4 Fig — The excimers/isoemisive ratio (474/432 nm) was followed at different temperatures during heating (A), or cooling (B) of the samples. CPPs were incubated with the LUVs at a 1/25 P/L ratio. Control CPP free LUVs black, Penetratin green, R9 blue and RW9 red. Means ± SEM of 4 to 8 independent experiments. The stars in colour correspond to their respective experimental point colours compared to the control LUVs; * P<0.05, ** P<0.01 by unpaired t-test. (JPG) [file pone.0210985.s004.jpg]

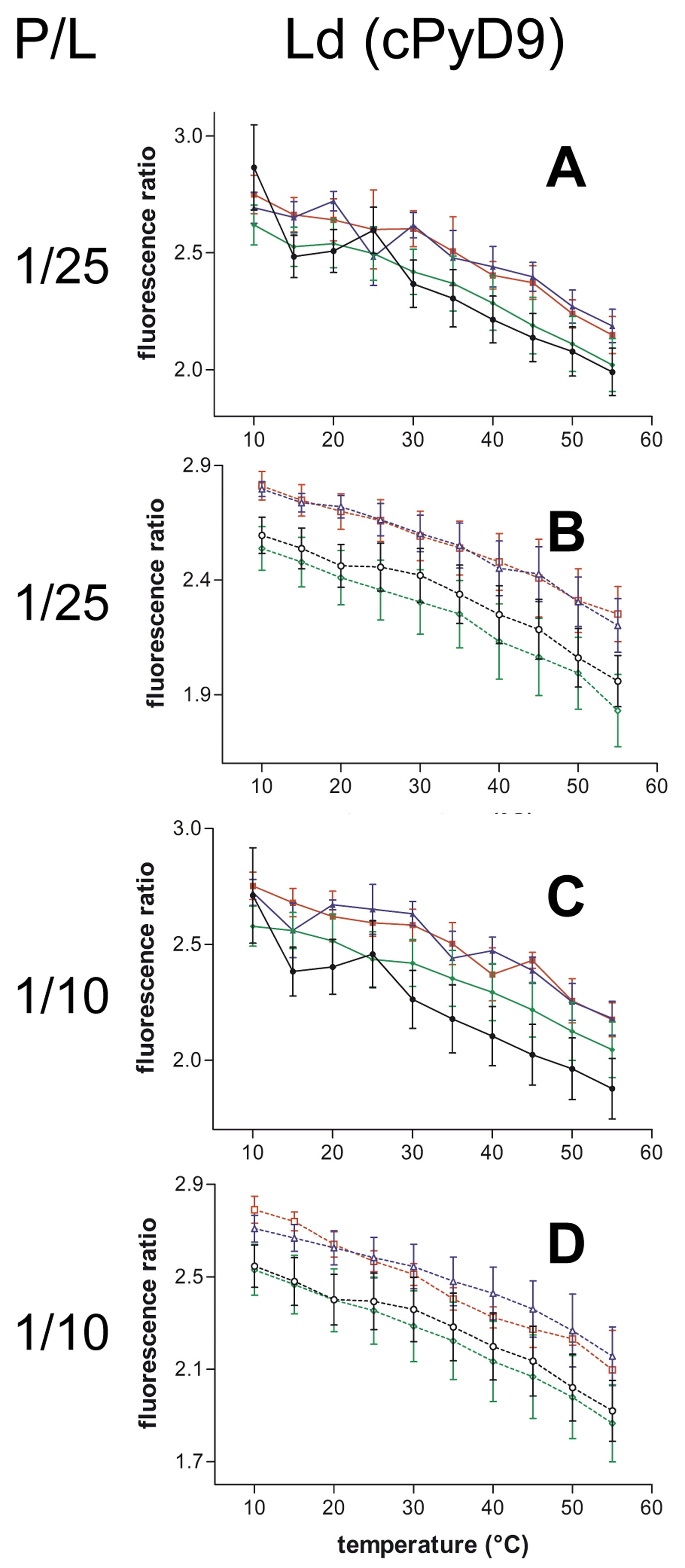

Supplement: S5 Fig — The 379/432 ratios were followed at different temperatures during heating (continuous lines), or cooling (dotted lines) of the samples. CPPs were incubated with the LUVs at a 1/25 P/L ratio (top panels) and 1/10 ratio (bottom panels). The Liquid disordered contribution (Ld) is quantified by the 379/432 nm ratio (cPyD9). Control CPP free LUVs black, Penetratin green, R9 blue and RW9 red. Means ± SEM of 4 to 9 independent experiments. (JPG) [file pone.0210985.s005.jpg]

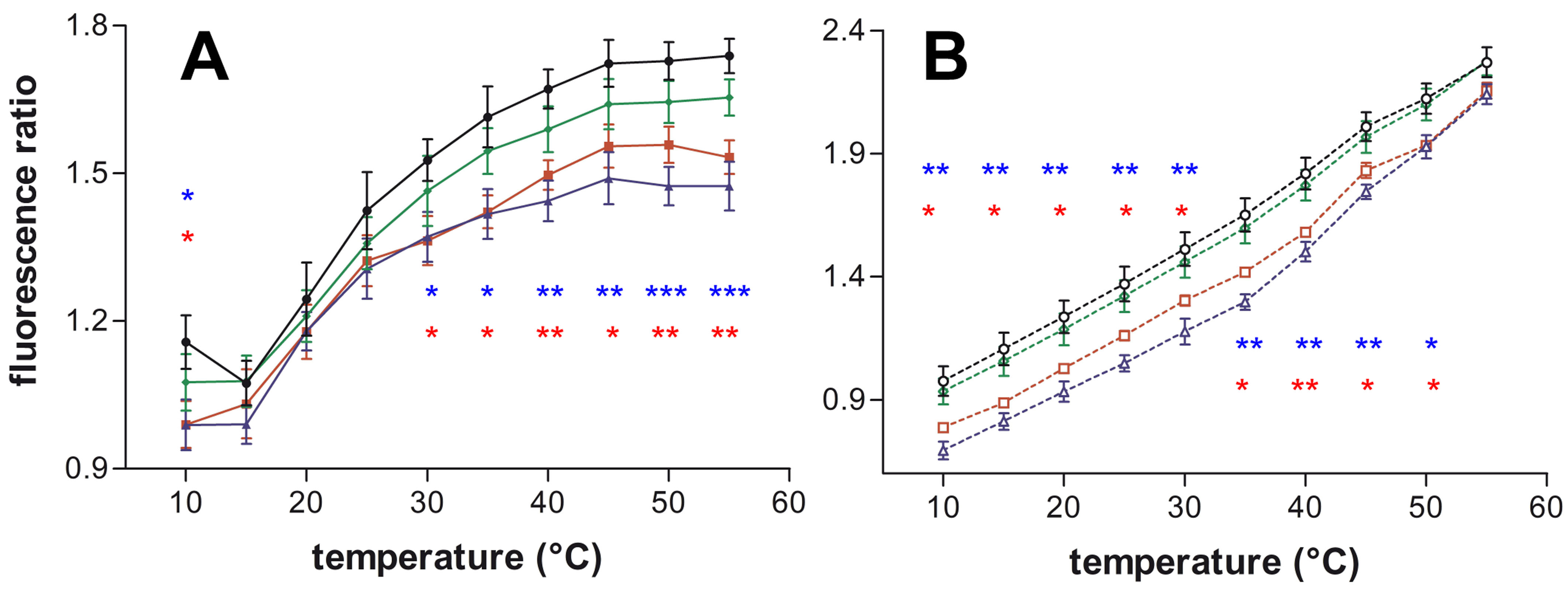

Supplement: S6 Fig — The excimers/isoemisive ratio (474/432 nm) was followed at different temperatures during heating (A), or cooling (B) of the samples. CPPs were incubated with the LUVs at a 1/25 P/L ratio. At a 1/10 P/L ratio the peptides effects on excimers were identical to those at 1/25 ratio. Control CPP free LUVs black, Penetratin green, R9 blue and RW9 red. Means ± SEM of 5 to 8 independent experiments. The stars in colour correspond to their respective experimental point colours compared to the control LUVs; * P<0.05, ** P<0.01, *** P<0.001 by unpaired t-test. (JPG) [file pone.0210985.s006.jpg]

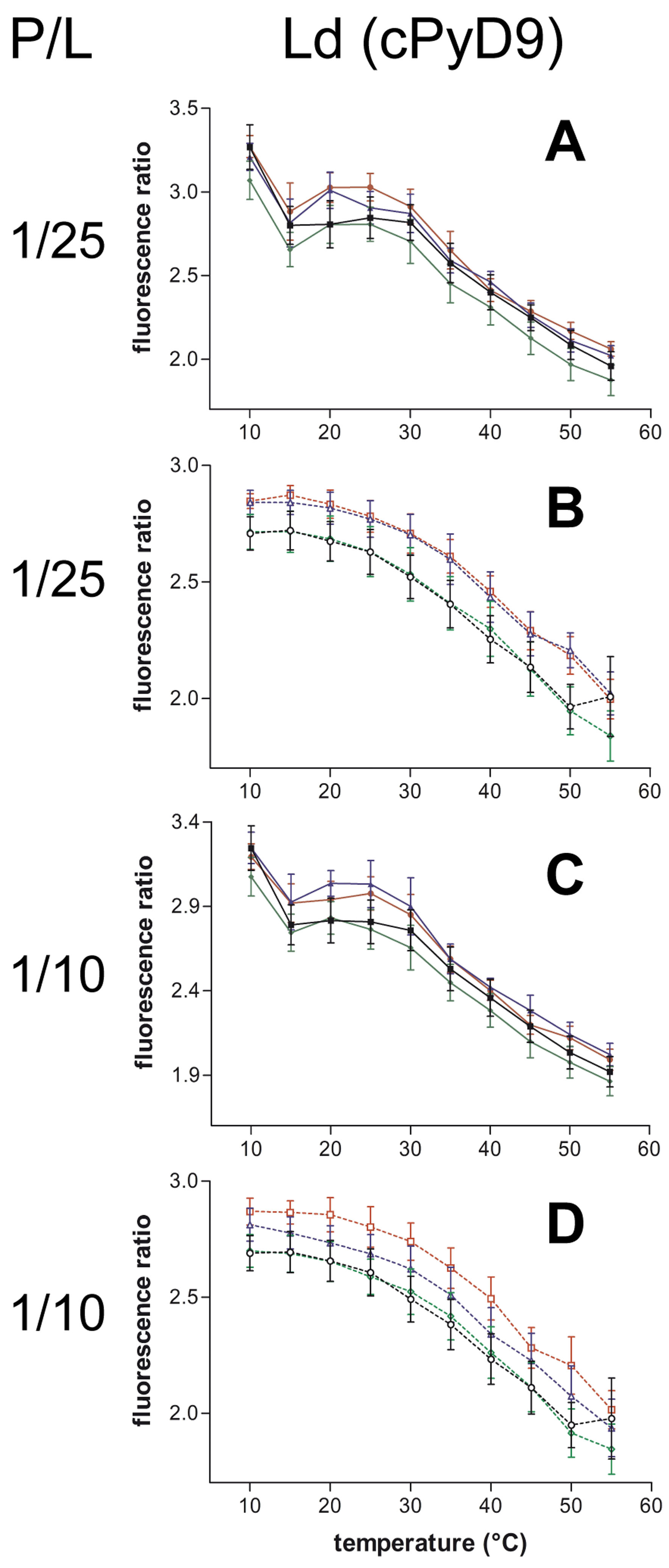

Supplement: S7 Fig — The different ratios were followed at 379/432 temperatures during heating (continuous lines), or cooling (dotted lines) of the samples. CPPs were incubated with the LUVs at a 1/25 P/L ratio (top panels) and 1/10 ratio (bottom panels). The Liquid disordered contribution (Ld) is quantified by the 379/432 nm ratio (cPyD9). Control CPP free LUVs black, Penetratin green, R9 blue and RW9 red. Means ± SEM of 5 to 8 independent experiments. (JPG) [file pone.0210985.s007.jpg]
